# Depressive Symptoms Associated with Peripheral Artery Disease and Predicting Mortality in Type 2 Diabetes

**DOI:** 10.3390/biomedicines12010029

**Published:** 2023-12-21

**Authors:** Yu-Hsuan Li, Yu-Cheng Cheng, Hsiu-Chen Liu, Junyi Wu, I-Te Lee

**Affiliations:** 1 Division of Endocrinology and Metabolism, Department of Internal Medicine, Taichung Veterans General Hospital, Taichung 40705, Taiwan; brightlight720720@gmail.com (Y.-H.L.); fererro1552@hotmail.com (Y.-C.C.); meganjunyiwu@gmail.com (J.W.); 2 School of Medicine, National Yang Ming Chiao Tung University, Taipei 11221, Taiwan; 3 Department of Computer Science & Information Engineering, National Taiwan University, Taipei 10617, Taiwan; 4 Institute of Biomedical Sciences, National Chung Hsing University, Taichung 40227, Taiwan; 5 Department of Nursing, Taichung Veterans General Hospital, Taichung 40705, Taiwan; jane@vghtc.gov.tw; 6 School of Medicine, Chung Shan Medical University, Taichung 40201, Taiwan

**Keywords:** ankle–brachial index, geriatric depression scale, depression, mortality, peripheral artery disease, type 2 diabetes

## Abstract

This retrospective cohort study aimed to assess the mortality risk in patients with type 2 diabetes mellitus (DM) by screening for depressive symptoms and peripheral artery disease (PAD). We enrolled patients aged ≥60 years who had undergone assessments of both the ankle–brachial index (ABI) and the five-item Geriatric Depression Scale (GDS-5). PAD and depression were defined as ABI ≤ 0.90 and GDS-5 ≥ 1, respectively. The primary endpoint was total mortality. In 1673 enrolled patients, the prevalence of PAD was higher in those with depression than in those without depression (8.9% vs. 5.7%, *p* = 0.021). After a median follow-up of 56.6 months (interquartile range: 47.0–62.3 months), a total of 168 (10.0%) deaths occurred. The patients in the depression and PAD subgroup had the highest hazard ratio of mortality, followed by the PAD without depression subgroup and the depression without PAD subgroup (2.209, 95%CI: 1.158–4.217; 1.958, 95%CI: 1.060–3.618; and 1.576, 95%CI: 1.131–2.196; respectively) in comparison to the patients without depression and PAD after adjustment for associated factors. In conclusion, a combination of depression and PAD predicted the highest mortality risk. Screening for depression and PAD is recommended in patients aged ≥60 years with type 2 DM.

## 1. Introduction

Depression has become an important health burden worldwide [1]. According to data from the Global Burden of Disease study between 1990 and 2017, the prevalence of depression has been increasing in the Chinese population aged over 55 years [2]. According to population-based data by questionnaire screening in the Healthy Aging Longitudinal Study, the prevalence of minor depression was 3.7%, and the prevalence of major depression was 1.5% in people aged over 55 years in Taiwan [3]. There is strong evidence that depression increases the risk of cardiovascular disease, disability, and mortality [2,4,5].

In particular, the presence of depressive symptoms is associated with an increased prevalence of diabetes mellitus (DM) [6]. Based on data from the UK Biobank, 5.5% of patients with type 2 DM developed major depression, and the risk, with a hazard ratio (HR) of 1.6, was significantly increased compared to patients without type 2 DM during a longitudinal follow-up for a median of 12.7 years; patients with type 2 DM also had a significantly higher risk of developing depressive symptoms than people without DM during a follow-up for a median of 7.5 years [7]. According to the National Health Insurance (NHI) research database regarding people in Taiwan between 2000 and 2007, the incidence of depression was 7.03 per 1000 person-years in patients with type 2 DM, and the HR of 1.43 was significantly higher than that in people without DM [8].

In addition to the association between the presence of DM and depression, there is a synergistic effect of depression and DM on mortality risk. Compared with people with neither DM nor depression, patients with a combination of DM and depression had an increased risk of mortality, and the corresponding HR of 2.16 was higher than that of patients with DM alone or depression alone according to the UK Biobank dataset for a median 6.8 years of follow-up [9]. Similarly, having a combination of DM and depressive symptoms is associated with a higher risk of mortality than DM alone or depression alone according to Taiwan population-based data for a longitudinal follow-up of 10 years [10]. The increased mortality risk might be contributed to the link between depression and cardiovascular risk in patients with type 2 DM [7].

Peripheral artery disease (PAD) is a well-known risk factor for cardiovascular events and mortality [11]. PAD is also a prevalent macrovascular complication in patients with DM [12], and DM was reported to confer a 1.4-fold increased risk of mortality in patients with PAD in a mean follow-up of 5.9 years [13]. However, the prevalence of PAD might be underestimated. Hong et al. [14] reported that only 34.7% of patients with an ankle–brachial index (ABI) ≤ 0.9 had been previously diagnosed with PAD based on administrative data. Measurement of the ABI can detect PAD early and is recommended by guidelines for clinical practice [15,16].

Depression is also an important risk factor for PAD. A meta-analysis study suggested that depression increases the mortality risk by 24% in patients with PAD [17]. However, depression might be underdiagnosed in patients with PAD. Welch et al. [18] reported that 28.4% of 148 patients undergoing interventions for PAD were diagnosed with depression based on screening with the 15-item Geriatric Depression Scale (GDS), but only 3.3% had a documented history of depression. Therefore, a simple questionnaire with high sensitivity is warranted to screen depression in patients at high risk. The five-item GDS (GDS-5), extracted from the 15-item GDS, has been developed and validated for screening depressive symptoms [19,20], but data on long-term mortality are scarce. We hypothesized that depressive symptoms are not only associated with PAD but are also predictive of long-term mortality in patients with type 2 DM. Therefore, we conducted a retrospective cohort study to assess the mortality risk categorized by GDS-5 and ABI in patients with type 2 DM.

## 2. Materials and Methods

### 2.1. Study Design and Population

This retrospective cohort study was conducted at Taichung Veterans General Hospital. The diabetic pay-for-performance (P4P) program has been an important policy launched by the NHI administration in Taiwan [21], and an annual comprehensive assessment including screening for PAD and mental health is recommended in this program [22]. In addition to ABI, the screening of GDS-5 has been included in the annual comprehensive assessment of diabetic P4P program in patients aged ≥60 years at Taichung Veterans General Hospital since 1 August 2016.

We screened candidates from outpatients of the Division of Endocrinology and Metabolism. The inclusion criteria were (1) individuals aged ≥60 years, (2) patients with DM, and (3) patients who had received assessments of both ABI and GDS-5 in the same annual comprehensive assessment between August 2016 and July 2020. The exclusion criteria were (1) current use of antidepressant or dementia drugs at the time of interview for answering the questionnaire, (2) DM other than type 2, (3) estimated glomerular filtration rate (eGFR) < 15 mL/min/1.73 m^2^, (4) unreliable ABI data, including ABI value ≥ 1.4, history of lower-limb surgery, or systolic blood pressure not detected in any of the four limbs, and (5) death within 30 days after enrollment.

### 2.2. Assessment of Risk Factors

The clinical data in the same annual comprehensive assessment were extracted from the electronic medical records. For patients who had undergone repeated comprehensive assessments during this study period, only the data from the first completed assessment were recorded. The characteristics of patients were age, sex, body height, body weight, blood pressure, smoking status, history of cardiovascular disease and hypertension, and current use of medication. The laboratory data comprised hemoglobin A1c (HbA1c), plasma glucose, serum levels of total cholesterol, high-density lipoprotein (HDL) cholesterol, triglycerides and creatinine, and urinary albumin and creatinine. HbA1c was measured using cation-exchange high-performance liquid chromatography (NGSP certified; G8, TOSOH, Tokyo, Japan). Serum lipid profiles and creatinine levels were measured by commercial kits (Beckman Coulter, Fullerton, CA, USA).

Overweight in Taiwan was defined as a body mass index (BMI) ≥ 24 kg/m^2^ [23]. Hypertension was defined as a systolic blood pressure ≥ 130 mmHg, a diastolic blood pressure ≥ 80 mmHg, or the current use of an antihypertensive drug. Low HDL cholesterol was defined as an HDL cholesterol level < 50 mg/dL (1.29 mmol/L) in women or <40 mg/dL (1.03 mmol/L) in men. The eGFR was calculated using the Chronic Kidney Disease Epidemiology Collaboration (CKD-EPI) equation for Chinese individuals with type 2 DM [24]. CKD was defined as an index eGFR < 60 mL/min/1.73 m^2^. The urinary albumin-to-creatinine ratio (UACR) was calculated using the ratio of urine albumin (mg/dL) to urine creatinine (g/dL), and increased albuminuria was defined as a UACR ≥ 30 mg/g. Diabetic kidney disease (DKD) was defined as CKD and/or increased albuminuria.

### 2.3. Assessments of GDS-5 and ABI

GDS-5 comprises 5 items reported by patients themselves. Item 1 received a score of 1 when a participant answered “No”, and Items 2 to 5 received a score of 1 when a participant answered “Yes”. The total score for GDS-5 ranges from 0 to 5, and a score ≥ 1 has a high sensitivity for the diagnosis of depression [19,20]. Therefore, depression is defined as a reported GDS-5 core ≥ 1 in the present study. The Mandarin Chinese version of GDS-5 has been reported in previous studies [25,26].

To measure ABI, blood pressure was detected at the bilateral brachial arteries and at the bilateral ankles using a validated automatic device (VP-1000 Plus; Omron healthcare Co. Ltd., Kyoto, Japan) after patients had rested in the supine position for at least 5 min. The ABI value was calculated as the ratio of the systolic blood pressure at each ankle to the higher systolic blood pressure of the bilateral brachial pressures [27]. The lower value of bilateral ABI in the same patient was recorded for the analyses. The reproducibility of ABI was reported in our previous study. Briefly, based on the Bland–Altman plots, the 95% confidence interval (CI) for the bias of ABI was 0.02 ± 0.01 between the repeated measurements in a group of 20 subjects [28]. PAD is defined as a recorded ABI value ≤ 0.90 [15,27].

### 2.4. Statistical Analysis

The total mortality served as the primary outcome. After collection of all the clinical data, the occurrence of mortality was recorded through 31 March 2022. Information on registered deaths was obtained from the Ministry of Health and Welfare, Executive Yuan, Taiwan. This research protocol was approved by the Institutional Review Board of Taichung Veterans General Hospital, and the need for informed consent was waived due to the retrospective cohort study design.

Continuous data are presented as the mean ± standard deviation, and categorical data are summarized as numbers with percentages (%). The statistical significance of differences in continuous data was examined using Student’s t tests between patients with and without any depressive symptom and by using one-way analysis of variance tests among the four study subgroups. The statistical significance of differences in categorized data was examined using chi-square tests.

The cumulative risk of total mortality was assessed using the Kaplan–Meier analysis; the log-rank test was used to examine the statistical significance of differences in survival rates among groups. Cox proportional hazards regression analysis was conducted to examine the independent predictors of mortality, and HR values with a 95% CI are presented. Because age and sex were important confounding factors, we included age, sex, and other assessed variables that were significantly associated with a GDS-5 score or PAD in the multivariable models. A two-sided *p* value < 0.05 indicated statistical significance. Statistical analysis was performed using SPSS v22.0 (IBM Corp., Armonk, NY, USA).

## 3. Results

**Figure 1:**
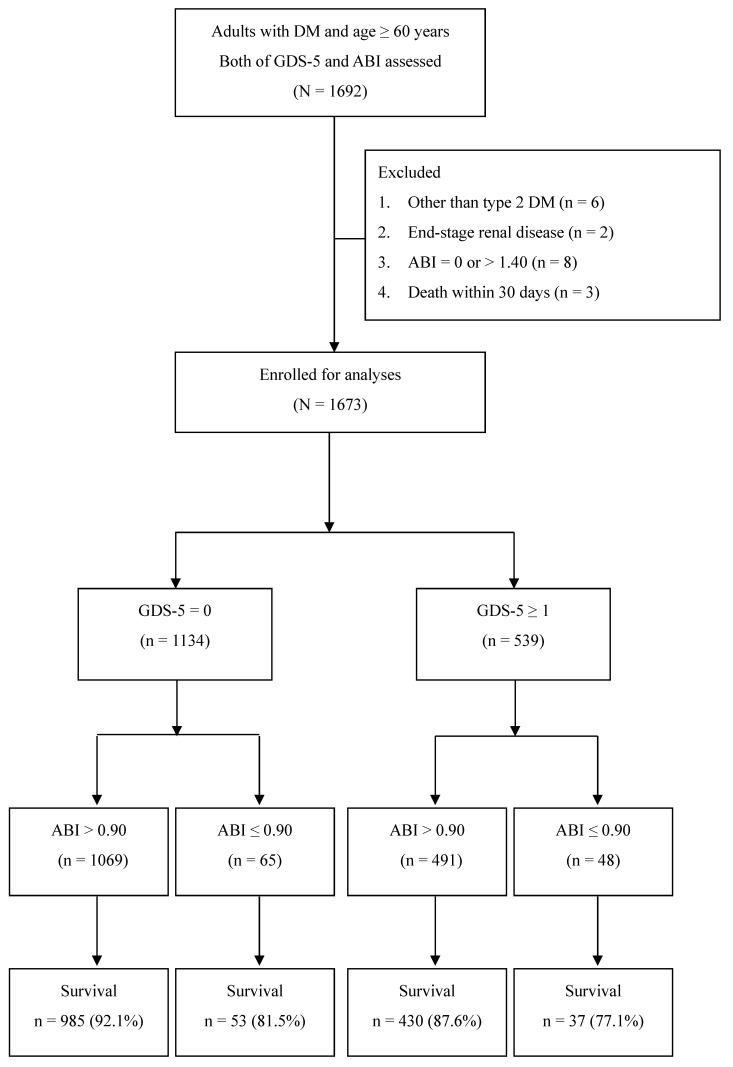
Flow diagram of the enrollment of study subjects (abbreviations: GDS-5, five-item Geriatric Depression Scale; ABI, ankle–brachial index; DM, diabetes mellitus).

In total, 1673 patients who met the study criteria were enrolled in this study (Figure 1). According to the self-reported GDS-5 questionnaire, 539 (32.2%) patients had at least one depressive symptom (GDS-5 ≥ 1), and 1134 patients had none of the five depressive symptoms (GDS-5 = 0). The baseline characteristics of patients with or without depressive symptoms are shown in Table 1. The patients with GDS-5 ≥ 1 had a significantly older age (73 ± 8 vs. 71 ± 7 years, *p* < 0.001) and significantly higher proportions of current smoking (8.3% vs. 4.9%, *p* = 0.009) and cardiovascular disease (27.8% vs. 20.7%, *p* = 0.002) than those with GDS-5 = 0. However, there was no significant difference in proportions of sex (*p* = 0.832), DKD (*p* = 0.999), hypertension (*p* = 0.181), and use of antihypertensive drugs (*p* = 0.167), antiplatelet drugs (*p* = 0.138), antidiabetic drugs (*p* > 0.05), and statins (*p* = 0.051) between patients with GDS-5 ≥ 1 and those with GDS-5 = 0. There was no significant difference in BMI (*p* = 0.664), systolic blood pressure (*p* = 0.152), diastolic blood pressure (*p* = 0.343), fasting plasma glucose (*p* = 0.653), HbA1c (*p* = 0.382), eGFR (*p* = 0.246), UACR (*p* = 0.062), and serum levels of total cholesterol (*p* = 0.618), HDL cholesterol (*p* = 0.426), triglycerides (*p* = 0.154), and ALT (*p* = 0.129) between patients with a GDS-5 ≥ 1 and those with GDS-5 = 0.

**Figure 2:**
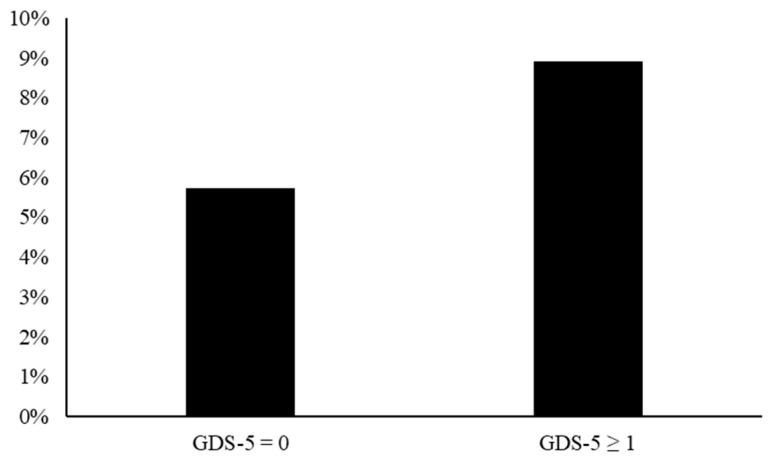
Prevalence of peripheral artery disease defined by ankle–brachial index ≤ 0.90 in the GDS-5 = 0 and GDS-5 ≥ 1 groups (8.9% vs. 5.7%, *p* = 0.021). (Abbreviation: GDS-5, five-item Geriatric Depression Scale).

Based on the definition of PAD as ABI ≤ 0.90, there were 113 (6.8%) patients with PAD in all enrolled patients. Notably, the ABI value was significantly lower in the patients with GDS-5 ≥ 1 than in those with GDS-5 = 0 (1.07 ± 0.13 vs. 1.09 ± 0.12, *p* = 0.007). The prevalence of PAD was also significantly higher in the patients with GDS-5 ≥ 1 than in those with GDS-5 = 0 (8.9% vs. 5.7%, *p* = 0.021, Figure 2).

During a median follow-up period of 56.6 months (interquartile range: 47.0–62.3 months), a total of 168 (10.0%) deaths occurred among all 1673 enrolled patients, including 72 in 539 patients (13.4%) with GDS-5 ≥ 1 and 96 in 1134 patients (8.5%) with GDS-5 = 0 (*p* = 0.002). The incidence of mortality was significantly higher in patients with GDS-5 ≥ 1 than in those with GDS-5 = 0 (3.1 vs. 1.9 per 100 person-years, log-rank test *p* = 0.002).

To assess the synergistic effect between GDS-5 and PAD on long-term mortality, we further divided all patients into four subgroups based on a cutoff value of 1 for GDS-5 and 0.90 for ABI. There were 1069 patients in the ABI > 0.90 and GDS-5 = 0 group, 491 patients in the ABI > 0.90 and GDS-5 ≥ 1 group, 65 patients in the ABI ≤ 0.90 and GDS-5 = 0 group, and 48 patients in the ABI ≤ 0.90 and GDS-5 ≥ 1 group. The baseline characteristics among these four groups are presented in Table 2. Patients with PAD and depression had a trend toward a higher proportion of age ≥ 70 years (*p* = 0.012), current smokers (*p* < 0.001), cardiovascular disease (*p* < 0.001), hypertension (*p* = 0.012), DKD (*p* < 0.001), CKD (*p* < 0.001), increased UACR (*p* < 0.001), and use of antiplatelet drugs (*p* < 0.001).

**Figure 3:**
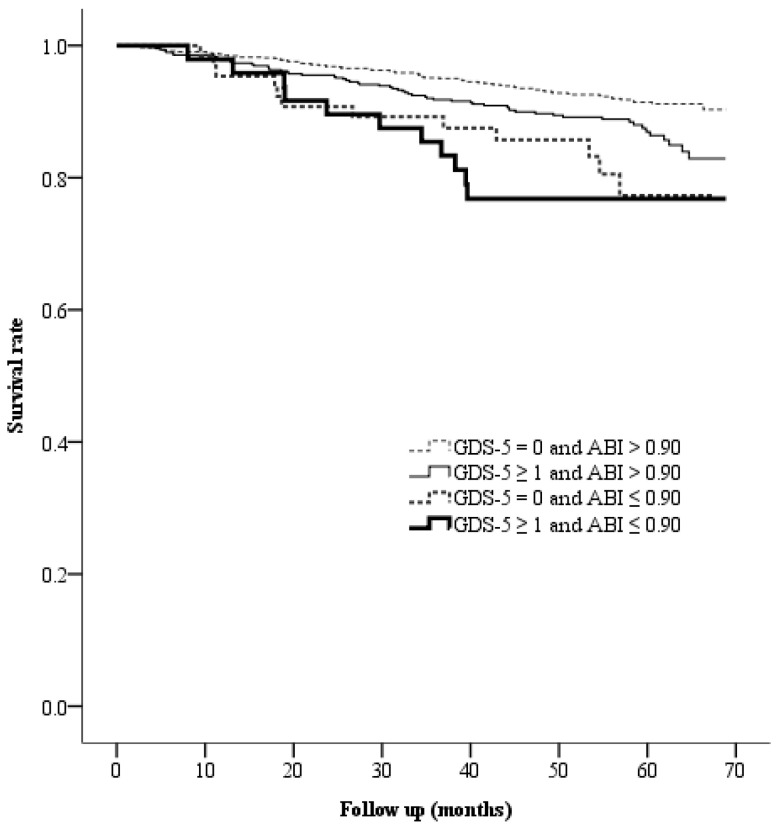
Kaplan–Meier curves presenting the survival rates across the four groups categorized based on GDS-5 and ABI. The median follow-up period was 56.6 months (interquartile range: 47.0–62.3 months). Abbreviations: GDS-5, five-item Geriatric Depression Scale; ABI, ankle–brachial index.

During the follow-up period, 84 (7.9%) deaths occurred in the ABI > 0.90 and GDS-5 = 0 group, 61 (12.4%) deaths occurred in the ABI > 0.90 and GDS-5 ≥ 1 group, 12 (18.5%) deaths occurred in the ABI ≤ 0.90 and GDS-5 = 0 group, and 11 (22.9%) deaths occurred in the ABI ≤ 0.90 and GDS-5 ≥ 1 group. The incidences of mortality were 1.8 per 100 person-years in the ABI > 0.90 and GDS-5 = 0 group, 2.8 per 100 person-years in the ABI > 0.90 and GDS-5 ≥ 1 group, 4.5 per 100 person-years in the ABI ≤ 0.90 and GDS-5 = 0 group, and 5.7 per 100 person-years in the ABI ≤ 0.90 and GDS-5 ≥ 1 group. The survival rates were significantly different among these four groups (log-rank test *p* < 0.001, Figure 3). In addition to age and sex, we selected the factors associated with GDS-5 and PAD, including smoking status, cardiovascular disease, hypertension, DKD, and use of antiplatelet drugs, which showed significant between-group differences (in Table 1 and Table 2) for the multivariable model. The mortality risks were still significantly different among these four groups after adjusting for the selected factors using multivariable Cox regression analysis. Compared to the mortality risk of the ABI > 0.90 and GDS-5 = 0 group, a significantly higher HR of 1.576 (95% CI: 1.131–2.196, *p* = 0.007) was observed in the ABI > 0.90 and GDS-5 ≥ 1 group, a significantly higher HR of 1.958 (95% CI: 1.060–3.618, *p* = 0.032) was observed in the ABI ≤ 0.90 and GDS-5 = 0 group, and the highest HR of 2.209 (95% CI: 1.158–4.217, *p* = 0.016) was observed in the ABI ≤ 0.90 and GDS-5 ≥ 1 group (Table 3). In the post hoc analyses between subgroups, patients in the ABI ≤ 0.90 and GDS-5 ≥ 1 group had a significantly higher mortality risk than those in the ABI > 0.90 and GDS-5 ≥ 1 group (HR = 1.924, 95% CI: 1.034–3.578; *p* = 0.039), but it was not significantly different from those in the ABI ≤ 0.90 and GDS-5 = 0 group (HR = 1.291, 95% CI: 0.577–2.892; *p* = 0.534) after adjusting for age and sex. Furthermore, there was no significant difference in the mortality risk between patients in the ABI > 0.90 and GDS-5 ≥ 1 group and those in the ABI ≤ 0.90 and GDS-5 = 0 group (*p* = 0.241).

## 4. Discussion

Our main finding in this study is that when depression was screened using GDS-5, it was significantly associated with PAD, which was screened using the ABI in patients with type 2 DM and aged ≥60 years. Patients with depression and PAD had the highest mortality risk, followed by those with PAD alone and those with depression alone, compared to those without depression or PAD during a median follow-up of 56.6 months. It is well known that depression is associated with mortality in patients with DM [9,10,29]. Depression has also been reported to be associated with mortality in patients with PAD [17]. The strength of the present study is that an increased risk of mortality could be predicted using both GDS-5 and ABI in patients aged ≥60 years with type 2 DM.

Only 2.7% of patients with DM had a diagnosis of depression in 2005 according to the international classification of diseases code based on the NHI research database [30], but the prevalence of depression might be 25.9% when screened by questionnaires in patients with type 2 DM and 29.5% in those aged ≥60 years based on meta-analyses of population-based studies, although the results were various and dependent on the questionnaire [31]. Based on the hospital-based population of the Indian Depression in Diabetes Study, the prevalence of depression was 35% in outpatients aged >60 years with type 2 DM according to questionnaire screenings [32]. In the present study, the prevalence of depression based on GDS-5 ≥ 1 was 32.2% in outpatients with type 2 DM and age ≥ 60 years. Compared to the 491 (31.5%) patients with depression in the 1560 patients without PAD, the prevalence of depression was significantly increased to 42.5% in the 113 patients with PAD.

PAD is prevalent in patients with depression [33]. Seldenrijk et al. [34] reported a higher prevalence of ABI < 0.9 in patients with an established diagnosis of depression than in those without depression; however, the prevalence of an ABI > 1.4 was not significantly associated with depression. Interestingly, Grenon et al. [33] reported that depressive symptoms were predictive of PAD development during a mean 7.2 years of follow-up, but the increased PAD incidence might be contributed to by comorbid cardiovascular risks in patients with depressive symptoms. However, in the prospective study of Atherosclerosis Risk in Communities (ARIC) with a mean 9.7 years of follow-up, a high depressive score was an independent predictor for PAD development after adjusting for cardiovascular risks [35]. McDermott et al. [36] also reported that patients with PAD developed more depressive symptoms assessed by GDS-15 than those without PAD during 2.7 years of follow-up. Therefore, the screening of ABI is suggested for patients with depressive symptoms, and vice versa.

It has been reported that the GDS-15 score is associated with mortality in older people [37]. In the present study, we detected depressive symptoms using GDS-5. Although a GDS-5 score cutoff value of 2 was suggested for depression diagnosis, a cutoff value of 1 can provide better sensitivity than other cutoff values [20], and we can further categorize the mortality risk by using ABI. There are several potential mechanisms involved in depression, PAD, and mortality in patients with type 2 DM. Depressive symptoms have been reported to be associated with chronic systemic inflammation reflected by an increase in inflammatory biomarkers in patients with PAD [38]. Depressive symptoms are also associated with the dysregulation of cortisol rhythm controlled by the hypothalamic–pituitary–adrenal axis [39]. The unsuppressed cortisol and autonomic dysfunction might induce insulin resistance and advanced atherosclerosis [40,41]. Moreover, ischemic heart disease and endothelial dysfunction are associated with a reduction in brain-derived neurotrophic factor (BDNF), which supports the functions and survival of neurons [42]. A reduction in BDNF, a bridge between atherosclerotic cardiovascular disease and depression [43], is predictive of long-term mortality [44]. Recently, a decrease in circulating BDNF was reported to be associated with CKD [45]. At present, DKD is not only a risk factor for dementia but also a predictor for mortality. BDNF, as a protective biomarker for depression, was shown to be a significant predictor for survival in patients with CAD and CKD [46]. Novak et al. [47] also reported that depression increased the risk of CKD and mortality.

In addition to CKD, age is a well-known factor for depression in community studies. In line with our study, Liu et al. [31] reported that a higher prevalence of depression was associated with older age in a population with type 2 DM. However, older age is also associated with more comorbidities. In the present study, the prevalence of depression was not significantly different between patients aged ≥70 years and <70 years (41.9% vs. 43.6%, *p* > 0.05) in the subgroup of patients with PAD. In contrast, in a hospital-based investigation, Majumdar et al. [32] reported that a higher prevalence of depression was associated with a younger age in outpatients with type 2 DM. Generally, female sex is a risk factor for depression [30,31,32]; however, there was no significant difference in depression prevalence between sexes in the present study. In line with our study, a hospital-based study reported that no significant difference in depression prevalence was observed between sexes in inpatients with type 2 DM [48]. According to the NHI research database, Chen et al. [22] reported that the proportion of female patients (52.3%) was higher than that of male patients with type 2 DM who were newly enrolled into the P4P program in 2004, but the trend of the female proportion in the P4P program decreased from 51.8% in 2005 to 50.0% in 2014 [21]. According to the NHI research database between 2005 and 2014, the mortality risk in male patients was higher than that in female patients with DM [49]. Similarly, male sex was still a significant predictor of mortality in the present study.

The categories of antidiabetic drugs were not significantly associated with depressive symptoms or PAD at baseline in the present study. Impaired insulin signaling in the brain plays a role in mood disorders in patients with type 2 DM [50]. As a technological improvement, intranasal delivery is a convenient method for providing insulin to the brain [51]. Despite an anxiolytic-like effect in mice [50], intranasal insulin delivery might not have a significant effect on depressive symptoms in the clinical study [52].

The present study has several limitations. First, all of the study data were collected from a single medical center. Second, we did not investigate the causal effects between PAD and depression. Third, we did not investigate the mechanisms of increased mortality risk caused by PAD and depression. Fourth, we only enrolled patients with type 2 DM in the P4P program, and the results could not be applied to other populations because it has been reported that the P4P program might reduce depression risk [53]. Fifth, we did not collect other demographic characteristics such as religion, education, occupation, and socio-economic level or personal habits such as alcohol intake and exercise. A lower socioeconomic status might be associated with not only depression but also PAD [54]. Finally, we only enrolled patients aged ≥60 years because the GDS-5 was clinically applied for screening in this population. The results of our study could not be applied to other age populations because the depressive risk might depend on age in patients with type 2 DM [31].

## 5. Conclusions

The use of GDS-5 and ABI is helpful for predicting long-term mortality in patients with type 2 DM aged ≥60 years in a regular diabetes management program. The prevalence of PAD was increased in patients with depressive symptoms, and vice versa. Screening of the self-report GDS-5 questionnaire and ABI is recommended in patients aged ≥60 years with type 2 DM. Further studies of early interventions in depression and PAD are warranted for patients with type 2 DM.

## Figures and Tables

**Table 1:** Baseline characteristics and mortality rate of enrolled patients categorized by GDS-5 score.

	GDS-5 = 0 (*n* = 1134)		GDS-5 ≥ 1 (*n* = 539)		*p*
Age (years)	71	± 7	73	± 8	<0.001
Male, *n* (%)	556	(49.0%)	268	(49.7%)	0.832
Current smoker, *n* (%)	56	(4.9%)	45	(8.3%)	0.009
CVD, *n* (%)	235	(20.7%)	150	(27.8%)	0.002
BMI (kg/m^2^)	25.4	± 3.8	25.3	± 4.0	0.664
Hypertension, *n* (%)	948	(83.6%)	465	(86.3%)	0.181
Systolic BP (mmHg)	138	± 20	139	± 20	0.152
Diastolic BP (mmHg)	75	± 10	76	± 11	0.343
Use of antihypertensive agents, *n* (%)	678	(59.8%)	342	(63.5%)	0.167
Fasting glucose (mmol/L)	7.7	± 2.5	7.8	± 2.6	0.635
HbA1c (%)	7.3	± 1.2	7.4	± 1.4	0.382
HbA1c (mmol/mol)	56.6	± 13.5	57.3	± 15.5	
Total cholesterol (mmol/L)	4.0	± 0.8	4.0	± 0.8	0.618
HDL cholesterol (mmol/L)	1.3	± 0.4	1.3	± 0.4	0.426
Triglycerides (mmol/L)	1.4	± 1.0	1.5	± 1.0	0.154
ALT (U/L)	24	± 17	25	± 19	0.129
Diabetic kidney disease, *n* (%)	604	(53.3%)	287	(53.2%)	0.999
eGFR (mL/min/1.73 m^2^)	67	± 15	66	± 15	0.246
UACR (mg/g)	184	± 554	247	± 795	0.062
ABI	1.09	± 0.12	1.07	± 0.13	0.007
Use of antiplatelet drugs, *n* (%)	363	(32.0%)	193	(35.8%)	0.138
Use of statins, *n* (%)	856	(75.5%)	382	(70.9%)	0.051
Use of antidiabetic drugs					
Insulin or insulin secretagogues, *n* (%)	656	(57.8%)	328	(60.9%)	0.265
Metformin, *n* (%)	400	(35.3%)	191	(35.4%)	0.992
Thiazolidinediones, *n* (%)	292	(25.7%)	120	(22.3%)	0.137
α-Glucosidase inhibitors, *n* (%)	131	(11.6%)	64	(11.9%)	0.912
DPP4 inhibitors, *n* (%)	733	(64.6%)	324	(60.1%)	0.082
SGLT2 inhibitors, *n* (%)	90	(7.9%)	49	(9.1%)	0.481
Mortality, *n* (%)	96	(8.5%)	72	(13.4%)	0.002

Continuous data are presented as the mean ± standard deviation, and categorical data are presented as numbers (percentages). Abbreviations: ABI = ankle–brachial index, ALT = alanine aminotransferase, BMI = body mass index, BP = blood pressure, CVD = cardiovascular disease, DPP4 = dipeptidyl peptidase-4, eGFR = estimated glomerular filtration rate, GDS-5 = five-item Geriatric Depression Scale, HbA1c = hemoglobin A1c, HDL = high-density lipo-protein, SGLT2 = sodium glucose cotransporter 2, UACR = urine albumin-to-creatinine ratio.

**Table 2:** Characteristics of enrolled patients categorized based on GDS and ABI.

	Group	*N*	ABI > 0.90 GDS-5 = 0 (*n* = 1069)		ABI > 0.90 GDS-5 ≥ 1 (*n* = 491)		ABI ≤ 0.90 GDS-5 = 0 (*n* = 65)		ABI ≤ 0.90 GDS-5 ≥ 1 (*n* = 48)		*p*
Age (years)	<70	797	534	(50.0%)	224	(45.6%)	22	(33.8%)	17	(35.4%)	0.012
	≥70	876	535	(50.0%)	267	(54.4%)	43	(66.2%)	31	(64.6%)	
Sex	Female	849	540	(50.5%)	251	(51.1%)	38	(58.5%)	20	(41.7%)	0.364
	Male	824	529	(49.5%)	240	(48.9%)	27	(41.5%)	28	(58.3%)	
Current smoker	No	1572	1020	(95.4%)	454	(92.5%)	58	(89.2%)	40	(83.3%)	<0.001
	Yes	101	49	(4.6%)	37	(7.5%)	7	(10.8%)	8	(16.7%)	
CVD	No	1288	853	(79.8%)	368	(74.9%)	46	(70.8%)	21	(43.8%)	<0.001
	Yes	385	216	(20.2%)	123	(25.1%)	19	(29.2%)	27	(56.3%)	
BMI (kg/m^2^)	<24	662	417	(39.0%)	201	(40.9%)	26	(40.0%)	18	(37.5%)	0.893
	≥24	1011	652	(61.0%)	290	(59.1%)	39	(60.0%)	30	(62.5%)	
Hypertension	No	243	170	(15.9%)	67	(13.6%)	2	(3.1%)	4	(8.3%)	0.016
	Yes	1430	899	(84.1%)	424	(86.4%)	63	(96.9%)	44	(91.7%)	
Systolic BP (mmHg)	<130	596	387	(36.2%)	181	(36.9%)	17	(26.2%)	11	(22.9%)	0.093
	≥130	1077	682	(63.8%)	310	(63.1%)	48	(73.8%)	37	(77.1%)	
Diastolic BP (mmHg)	<80	1167	747	(69.9%)	347	(70.7%)	44	(67.7%)	29	(60.4%)	0.509
	≥80	506	322	(30.1%)	144	(29.3%)	21	(32.3%)	19	(39.6%)	
Fasting glucose (mmol/L)	<7.2	802	506	(47.3%)	240	(48.9%)	36	(55.4%)	20	(41.7%)	0.470
	≥7.2	871	563	(52.7%)	251	(51.1%)	29	(44.6%)	28	(58.3%)	
HbA1c	<7%	739	476	(44.5%)	218	(44.4%)	24	(36.9%)	21	(43.8%)	0.693
	>7%	934	593	(55.5%)	273	(55.6%)	41	(63.1%)	27	(56.3%)	
Total cholesterol (mmol/L)	<4.1	1029	661	(61.8%)	297	(60.5%)	37	(56.9%)	34	(70.8%)	0.457
	≥4.1	644	408	(38.2%)	194	(39.5%)	28	(43.1%)	14	(29.2%)	
Low HDL cholesterol	No	1086	696	(65.1%)	327	(66.6%)	35	(53.8%)	28	(58.3%)	0.169
	Yes	587	373	(34.9%)	164	(33.4%)	30	(46.2%)	20	(41.7%)	
Triglycerides (mmol/L)	<1.7	1231	807	(75.5%)	349	(71.1%)	41	(63.1%)	34	(70.8%)	0.059
	≥1.7	442	262	(24.5%)	142	(28.9%)	24	(36.9%)	14	(29.2%)	
Diabetic kidney disease	No	782	514	(48.1%)	239	(48.7%)	16	(24.6%)	13	(27.1%)	<0.001
	Yes	891	555	(51.9%)	252	(51.3%)	49	(75.4%)	35	(72.9%)	
eGFR (mL/min/1.73 m^2^)	≥60	1130	739	(69.1%)	336	(68.4%)	32	(49.2%)	23	(47.9%)	<0.001
	<60	543	330	(30.9%)	155	(31.6%)	33	(50.8%)	25	(52.1%)	
UACR (mg/g)	<30	980	648	(60.6%)	292	(59.5%)	24	(36.9%)	16	(33.3%)	<0.001
	≥30	693	421	(39.4%)	199	(40.5%)	41	(63.1%)	32	(66.7%)	
ALT (U/L)	<20	806	520	(48.6%)	222	(45.2%)	33	(50.8%)	31	(64.6%)	0.067
	≥20	867	549	(51.4%)	269	(54.8%)	32	(49.2%)	17	(35.4%)	
Use of antiplatelet	No	1117	744	(69.6%)	328	(66.8%)	27	(41.5%)	18	(37.5%)	<0.001
	Yes	556	325	(30.4%)	163	(33.2%)	38	(58.5%)	30	(62.5%)	
Use of statins	No	435	267	(25.0%)	146	(29.7%)	11	(16.9%)	11	(22.9%)	0.067
	Yes	1238	802	(75.0%)	345	(70.3%)	54	(83.1%)	37	(77.1%)	
Use of antidiabetic drugs											
Insulin or insulin secretagogues	No	689	454	(42.5%)	196	(39.9%)	24	(36.9%)	15	(31.3%)	0.321
	Yes	984	615	(57.5%)	295	(60.1%)	41	(63.1%)	33	(68.8%)	
Metformin	No	1082	691	(64.6%)	312	(63.5%)	43	(66.2%)	36	(75.0%)	0.461
	Yes	591	378	(35.4%)	179	(36.5%)	22	(33.8%)	12	(25.0%)	
Thiazolidinediones	No	1261	794	(74.3%)	380	(77.4%)	48	(73.8%)	39	(81.3%)	0.432
	Yes	412	275	(25.7%)	111	(22.6%)	17	(26.2%)	9	(18.8%)	
α-Glucosidase inhibitors	No	1478	944	(88.3%)	434	(88.4%)	59	(90.8%)	41	(85.4%)	0.856
	Yes	195	125	(11.7%)	57	(11.6%)	6	(9.2%)	7	(14.6%)	
DPP4 inhibitors	No	616	380	(35.5%)	199	(40.5%)	21	(32.3%)	16	(33.3%)	0.215
	Yes	1057	689	(64.5%)	292	(59.5%)	44	(67.7%)	32	(66.7%)	
SGLT2 inhibitors	No	1534	981	(91.8%)	446	(90.8%)	63	(96.9%)	44	(91.7%)	0.421
	Yes	139	88	(8.2%)	45	(9.2%)	2	(3.1%)	4	(8.3%)	

ABI = ankle–brachial index, ALT = alanine aminotransferase, BMI = body mass index, CVD = cardiovascular disease, DPP4 = dipeptidyl peptidase-4, eGFR = estimated glomerular filtration rate, GDS-5 = five-item Geriatric Depression Scale, HbA1c = hemoglobin A1c, HDL = high-density lipoprotein, SGLT2 = sodium glucose cotransporter 2, UACR = urine albumin-to-creatinine ratio.

**Table 3:** Cox regression analysis for the risk of mortality.

	Crude				Model 1				Model 2			
	HR	95%	CI	*p*	HR	95%	CI	*p*	HR	95%	CI	*p*
GDS-5 = 0 and ABI > 0.90	1.000				1.000				1.000			
GDS-5 ≥ 1 and ABI > 0.90	1.628	(1.171,	2.264)	0.004	1.558	(1.120,	2.167)	0.008	1.576	(1.131,	2.196)	0.007
GDS-5 = 0 and ABI ≤ 0.90	2.604	(1.422,	4.770)	0.002	2.238	(1.220,	4.105)	0.009	1.958	(1.060,	3.618)	0.032
GDS-5 ≥ 1 and ABI ≤ 0.90	3.294	(1.756,	6.177)	<0.001	2.758	(1.469,	5.178)	0.002	2.209	(1.158,	4.217)	0.016
Age ≥ 70 years					3.245	(2.257,	4.666)	<0.001	2.519	(1.733,	3.662)	<0.001
Male					1.776	(1.302,	2.424)	<0.001	1.488	(1.074,	2.062)	0.017
Current smoker									0.942	(0.502,	1.768)	0.853
Cardiovascular disease									1.442	(0.991,	2.096)	0.056
Hypertension									1.501	(0.840,	2.680)	0.170
Diabetic kidney disease									2.469	(1.656,	3.680)	<0.001
Use of antiplatelet drugs									0.826	(0.575,	1.185)	0.299

Abbreviations: ABI = ankle–brachial index, CI = confidence interval, GDS = five-item Geriatric Depression Scale.

## Data Availability

The data presented in this study are available on request from the corresponding author.
